# Smallpox in Texas

**Published:** 1899-09

**Authors:** 


SMALLPOX IN TEXAS.—It is a reproach to the State that this foul disease has become almost perennial in Texas. There is nearly always an epidemic more or less extensive in some part or parts of the State, and the State Health Officer is kept constantly running about and giving instructions as to "stamping it out." Just how much longer a civilized people are going to expend time, money and energy in thus dealing with results of causes easily and demonstrably preventable, instead of rationally directing those efforts to *prevention,* is a question. Judging from the negative results that have attended every effort of the medical profession of Texas to impress the law-making power of the State and its executive with the importance, the necessity of sanitary laws that shall reverse the order, and deal with the causes of disease, it will be many generations. For twenty years the medical profession have been preaching the gospel of sanitation; but their sermons have fallen on ears too much occupied with political schemes; while the medical press of the State have wrung all the changes on the subject, vainly arguing that "to be sanitary is to be sane;" but the trouble has been, that the medical press has no audience except medical men; the secular press as a rule will not, or do not reproduce the arguments, and the people are living on in blissful ignorance of the dangers that beset them at every turn, and have no conception of the fact that death in the family, and doctor's bills, and drug bills, are the result of such ignorance on their part, and of causes which could easily be removed if an enlightened public sentiment were engendered. It is an old and homely saying that "you may take a horse to water, but you can't make him drink." You may preach sanitary reform; you may parade statistics to show the splendid results obtained in other communities by such reform, and may send marked copies of such articles to governors and legislators, but no power on earth can make them read them. The Japanese who, less than fifty years ago were using bows-and-arrows in war, are shaming civilized Texas in sanitary progress. The Japanese government is enforcing compulsory vaccination. The JOURNAL has urged the necessity of some such measure in Texas till the subject is well-nigh threadbare. We again insist upon it as the only rational means of dealing with smallpox, and although we don't suppose that it will have any more influence with the governor of Texas or the legislators—if they ever see it or read it—than have other statistics we have published, we give below the figures to show what compulsory vaccination has recently done in Germany.

"The latest reports concerning smallpox in Germany during the last decade and a half show that for the ten years previous to 1895 the average death rate from smallpox was 116, the greater of the total number occurring in the early period of the decade. In 1895 there were 27 deaths, in 1896, 10, and in 1897, 5. In Germany the law requires vaccination and revaccination, and the law is carried out. In no other country is the value of vaccination more evident than in Germany, and in no other country is the preventive method more thoroughly carried out."

